# Sex differences in the influence of cognitive reserve factors on late‐life cognition

**DOI:** 10.1002/alz70860_107137

**Published:** 2025-12-23

**Authors:** Catie J Wandell, Sarah Schwartz, Gail B. Rattinger, JoAnn T. Tschanz

**Affiliations:** ^1^ Utah State University, Logan, UT, USA; ^2^ Binghamton University, Binghamton, NY, USA

## Abstract

**Background:**

Despite increased dementia incidence in women and the gendered nature of many known cognitive reserve (CR) factors, few studies have examined sex differences in CR on late‐life cognition. How gendered CR factors, such as occupation and child rearing, differentially affect cognition in older adults remains unclear. We examined sex differences in the contribution of gendered CR factors on late‐life cognitive decline.

**Method:**

Participants were 4375 older adults (57% women, M(SD) age = 74.82(6.75) years) of the Cache County Memory Study, followed for a maximum of 11.39 years. The Modified Mini‐Mental State (3MS) assessed cognition across four triennial waves. Demographics, number of offspring (women only), and occupational history were obtained. Occupational attainment was classified as: 1) Professional‐Technical‐Managerial, 2) Clerical‐Sales, 3) Service, 4) Agriculture‐Natural Resources, 5) Processing‐Machine trades‐Bench‐Structural work‐Miscellaneous, and 6) Never Employed. For women, categories 4 and 5 were combined due to sparseness. The associations between number of offspring, occupational attainment and baseline marital status with cognitive decline were examined using linear mixed effects models, controlling for age and education.

**Result:**

All men reported a history of employment; 14.6% of women reported never being employed. Those in professional‐technical‐managerial occupations obtained the highest 3MS scores in both sexes. Among men, service workers scored lower on the 3MS (mean differences 1.8 – 3.2 points) than all other occupations, with the exception of agriculture‐natural resources (*p*'s < .007) whereas among women, service workers scored lower than those in professional‐technical‐managerial, clerical‐sales positions, and those who never worked (mean differences 1.3 – 1.8 points; *p*'s < .001). Notably, women who never worked did not score significantly lower than women with an employment history. Cognitive scores did not differ for women with or without offspring (*p*'s > .20), whereas widowed women experienced more rapid decline than their married counterparts (b = ‐.44, *p* < .001). Marital status was not associated with late‐life cognition in men.

**Conclusion:**

Our analyses suggest that late‐life cognition in men and women is differentially affected by gendered lifespan CR factors. In future work, we will explore sex differences in CR factors accumulated across the lifespan to elucidate interactive effects of early vs. late‐life experiences on cognition.

